# Wolf habitat selection when sympatric or allopatric with brown bears in Scandinavia

**DOI:** 10.1038/s41598-020-66626-1

**Published:** 2020-06-18

**Authors:** Andrés Ordiz, Antonio Uzal, Cyril Milleret, Ana Sanz-Pérez, Barbara Zimmermann, Camilla Wikenros, Petter Wabakken, Jonas Kindberg, Jon E. Swenson, Håkan Sand

**Affiliations:** 1 Faculty of Applied Ecology, Agricultural Sciences and Biotechnology, Inland Norway University of Applied Sciences, Evenstad, NO-2480 Koppang, Norway; 2 Faculty of Environmental Sciences and Natural Resource Management, Norwegian University of Life Sciences, Post box 5003, NO–1432 Ås, Norway; 3 School of Animal, Rural and Environmental Sciences, Nottingham Trent University, Brackenhurst, Southwell, Nottinghamshire NG25 0FQ UK; 4 Biodiversity and Animal Conservation Lab, Forest Science and Technology Centre of Catalonia (CTFC), 25280 Solsona, Spain; 5 Grimsӧ Wildlife Research Station, Department of Ecology, Swedish University of Agricultural Sciences, SE-730 91 Riddarhyttan, Sweden; 6 Norwegian Institute for Nature Research, NO–7485 Trondheim, Norway; 7 Department of Wildlife, Fish, and Environmental Studies, Swedish University of Agricultural Sciences, SE–901 83 Umea, Sweden

**Keywords:** Behavioural ecology, Animal behaviour

## Abstract

Habitat selection of animals depends on factors such as food availability, landscape features, and intra- and interspecific interactions. Individuals can show several behavioral responses to reduce competition for habitat, yet the mechanisms that drive them are poorly understood. This is particularly true for large carnivores, whose fine-scale monitoring is logistically complex and expensive. In Scandinavia, the home-range establishment and kill rates of gray wolves (*Canis lupus*) are affected by the coexistence with brown bears (*Ursus arctos*). Here, we applied resource selection functions and a multivariate approach to compare wolf habitat selection within home ranges of wolves that were either sympatric or allopatric with bears. Wolves selected for lower altitudes in winter, particularly in the area where bears and wolves are sympatric, where altitude is generally higher than where they are allopatric. Wolves may follow the winter migration of their staple prey, moose (*Alces alces*), to lower altitudes. Otherwise, we did not find any effect of bear presence on wolf habitat selection, in contrast with our previous studies. Our new results indicate that the manifestation of a specific driver of habitat selection, namely interspecific competition, can vary at different spatial-temporal scales. This is important to understand the structure of ecological communities and the varying mechanisms underlying interspecific interactions.

## Introduction

Habitat selection takes place at several spatial scales, typically described as four orders of selection^1^. Populations have a distribution range (1^st^ order); individuals establish home ranges (2^nd^), select habitats inside them (3^rd^), and use specific patches for specific activities, e.g., feeding and resting (4^th^). Habitat selection within home ranges depends on factors such as prey availability, landscape features, and intra- and interspecific interactions, which can reduce the selection of otherwise preferred habitats^2–4^. Thus, individual space use is influenced by interactions with other animals and the environment, highlighting the need to jointly investigate the population-level and spatial selection processes underlying such interactions^5,6^.

Interspecific competition influences the population dynamics and community structure of large carnivores^7^. Competition triggers different behavioral responses, including varying degrees of spatio-temporal overlap and avoidance among competitors, yet the behavioral mechanisms that drive carnivore responses to competition are poorly understood^8^. This topic is currently receiving increasing attention for different carnivore guilds in different continents^8–13^. However, the spatio-temporal dynamics of apex predator interactions still requires a better understanding^14^.

In northern Europe, the gray wolf (*Canis lupus*), brown bear (*Ursus arctos*), Eurasian lynx (*Lynx lynx*), and wolverine (*Gulo gulo*) constitute the large carnivore guild. Previous research on interspecific competition focused on their habitat and resource use^15–17^, interference competition between trophic levels^18,19^, and the demographic impact of coexisting predators on prey^20,21^. The latter topic has management implications, e.g., to adjust harvest quotas of ungulates in areas with coexisting bears and wolves^22^.

In Scandinavia (Sweden and Norway), where wolves and bears are forest-living species^11^, moose constitutes the main prey for wolves all year long, and for bears in spring^23,24^, which can trigger interactions, e.g., via scavenging of the kills made by each other^25,26^. The establishment of wolf pairs during the ongoing wolf population recovery, i.e., wolf 2^nd^ order habitat selection, has been affected positively by previous wolf presence and negatively by road density, distance to other wolf territories, and bear density^26,27^. Proximity to other wolf territories is a proxy of density, which is a major driver of habitat selection^28^, and road density reflects anthropogenic habitat encroachment, which is also well known as a driver of large carnivore behavior^29^. However, the negative effect of bear density on the probability of establishment of wolf pairs was a novel finding regarding the effects that interspecific competition between apex predators can have at the population level^26,27^.

The role of influential factors can vary at different orders of habitat selection, both spatially and temporally^2,30,31^. For instance, habitat selection of sympatric bobcats (*Lynx rufus*) and coyotes (*Canis latrans*) differed slightly at the landscape scale, but not within home ranges^32^. Seasonal- and scale-related differences have also been shown for ungulates^33^, and in specific habitat-selection studies of wolves^34^ and brown bears^35,36^. In Scandinavia, we have also found that, within home ranges, sympatric wolves and bears segregate more than expected by chance in their habitat selection^37^, and wolf kill rates are lower when they are sympatric with bears than when not sympatric^38^. That bear presence triggers different responses of wolves at different spatial scales can be interpreted as a reinforcement of a pattern of behavior^30^.

To further understand the effects of interspecific competition on large carnivore habitat selection at different spatial contexts, we analyzed the potential variation in wolf habitat selection within home ranges (3^rd^ order of habitat selection) of adult territorial individuals throughout the breeding range of the species in Scandinavia, i.e., when sympatric or allopatric with bears. Based on previous results^26,27,37,38^, we predicted differences in wolf habitat selection when sympatric compared to allopatric with bears. Because all Scandinavian bears hibernate during winter^39^, these differences would occur in summer, rather than in winter. Beyond wolves and bears, understanding factors that shape apex predators’ co-occurrence at different scales would help advance our knowledge of the structure of biotic communities.

## Methods

### Study area

**Figure 1:**
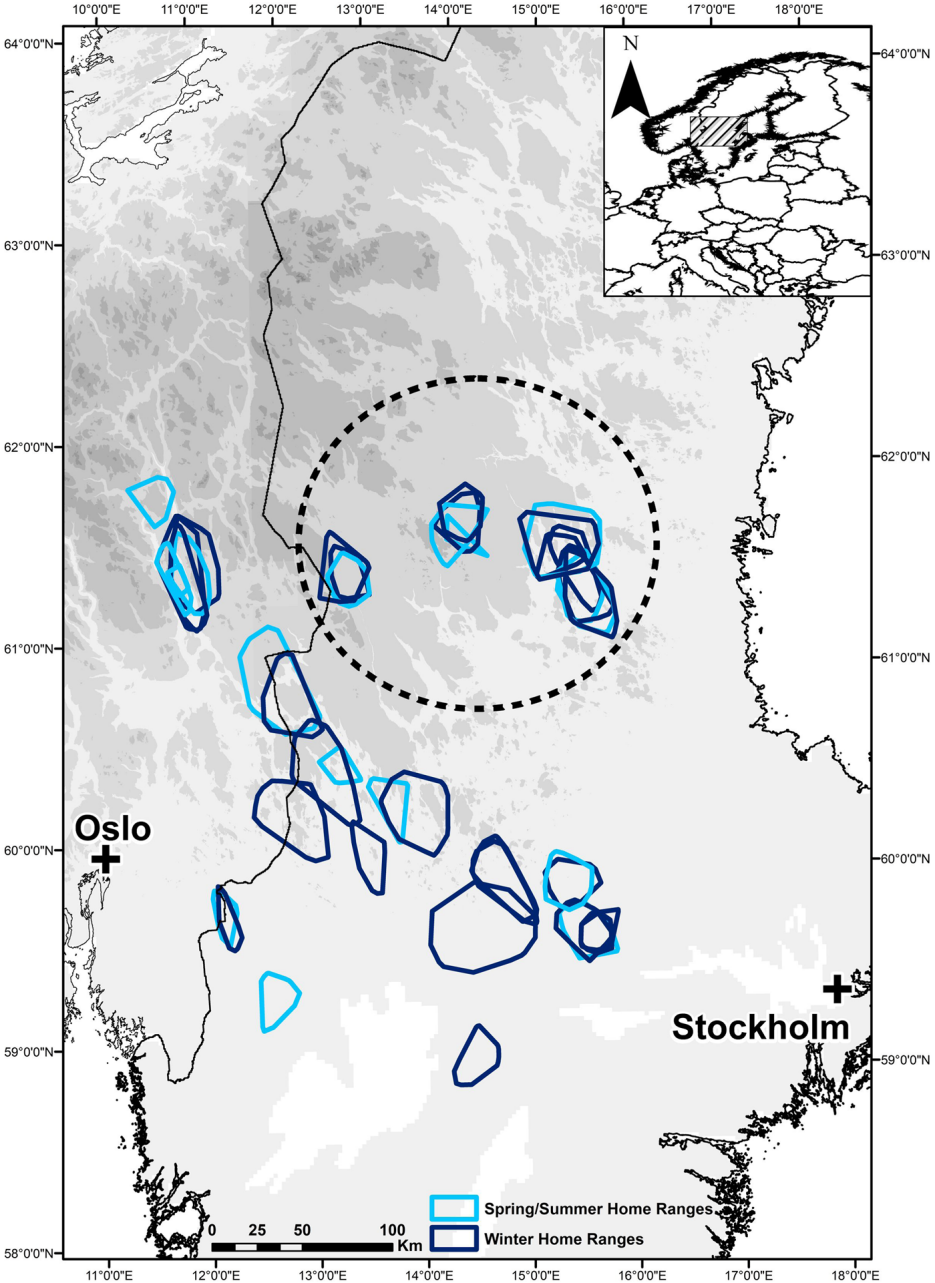
Distribution of wolf territories (100% Minimum Convex Polygon) sympatric (within the highlighted circle) or allopatric with brown bears in Scandinavia during 2001–2016. The median elevation range (higher elevation represented by darker background) was typically higher for the northern wolf territories that were sympatric with bears than for the allopatric wolf territories in the south (see Methods). Winter wolf home ranges are displayed in dark blue and spring/summer home ranges in lighter blue. White areas in the lower part of the map represent the biggest lakes in south-central Sweden.

The breeding range of wolves, approx. 100,000 km^2^ in southcentral Scandinavia, defined our study area (Fig. 1). It consists of an intensively managed coniferous boreal forest, intersected with bogs and lakes. Agricultural land is more common in the south, west, and east of the area. Altitudes range from ~50 to 1000 m above sea level. The density of primary roads within the wolf range is 0.2 + 0.02 km/km^2^, gravel road density is 4.6 times higher^40^, and the human population density is low, with <1 person/km^2^ over large areas^26^. The wolf population size was ~410 wolves (95% CI 324–533) in the winter of 2017/2018^41^, and average home range size of wolves in Scandinavia is approximately 1000 km^2^^42^. Wolf packs are sympatric with bears only in the northern part of the study area (61°N, 15°E; Fig. 1). Since 2000/2001, one to eight wolf packs have been recorded annually within the bear range in central Sweden^37^. Moose densities in Scandinavia are among the highest in the world (~0.5–2 moose per km^2^) and are the staple prey for wolves, with roe deer (*Capreolus capreolus*) as secondary prey^43^. The brown bear population (~2750 bears in 2013) reaches a density of 30 bears/1000 km^2^ in areas sympatric with wolves^44^. For context, annual home ranges of adult male bears average ~1000 km^2^ in the bear core area, five times larger than the ~200 km^2^ of adult females^45^. In early summer, neonate moose calves are a primary food item for brown bears in the study area, with most of the predation occurring in May and June^23^.

### Wolf data and overlap with brown bears

At least one scent-marking adult wolf in each of the studied wolf territories was radio-collared during the last 20 years, to address research and management questions^46^. For this study, we used data from 25 wolves in 22 wolf territories (44 wolf territory year/seasons), from 2001 to 2016. Wolves were captured with veterinary procedures approved by ethical committees (in Sweden, *Djurförsöksetiska nämnd*; in Norway, *Forsøksdyrutvalget*) and the wildlife management authorities (in Sweden, *Naturvårdsverket*; in Norway, *the Norwegian Environment Agency*), as described in Arnemo *et al*.^47^, i.e., captures were performed in accordance with relevant guidelines and regulations. Wolves were equipped with GPS–GSM neck collars (Followit Sweden AB, and VECTRONIC Aerospace GmbH, Germany). Collars recorded locations at 60- or 30-min intervals in late winter/early spring and late spring/early summer, primarily to conduct predation studies^38^. We did not subset all locations to the same fix interval, because locations were recorded at regular intervals for a given individual and season (either 60 or 30 min) and were therefore proportional to habitat use. Preliminary analyses documented that different fix intervals did not affect the main results. Usually, GPS receivers have an accuracy within 5 m of the actual position under open sky conditions and within 10 m under closed canopies^29^. We used the distribution of 3083 brown bear deaths, primarily caused by legal hunting, between 1990 and 2012 to create an index of bear density across Scandinavia^26^. The index was either very close to 0, i.e., in areas with no bears or sporadic bear presence, or >0.8 in areas with the highest bear density, which allowed us to categorize wolf territories as ‘allopatric’ or ‘sympatric’ with bears, respectively (Fig. 1)^26,38^. We studied wolf habitat selection at the scale of the wolf territory level for territories that were sympatric (N = 15; 9 in winter, 6 in spring-summer) or allopatric (N = 29; 17 in winter, 12 in spring-summer) with brown bears. The elevation range for the centroid of wolf territories sympatric with bears was 194–582 (median = 309) m.a.s.l., whereas it was 34–816 (median = 252) m.a.s.l., for allopatric wolf territories (Fig. 1).

### Wolf habitat selection

First, we estimated the home ranges of each wolf territory. Seasonality is important for wolf habitat selection^37^, thus we differentiated winter (1 December-30 April) and spring/summer (1 May-31 July) habitat selection. Because the periods with intensive GPS locations were limited to late winter, spring, and early summer (for the rest of the year, intervals between consecutive positions were longer to extend the collars’ battery life), the true home range sizes were likely somewhat underestimated^42^. We therefore defined availability by creating a 100% minimum convex polygon (MCP), for each season and year and for each wolf territory. We then sampled random locations (10 times the number of locations used by the wolves) to define habitat availability within the MCP for each wolf territory. To test the influence of the method chosen to define availability, we also performed the same analysis using 99% kernel home ranges, using the “href” method^48^. We extracted the habitat characteristics of used and available locations using habitat variables that are known to influence wolf habitat selection, i.e., forest cover, elevation, terrain ruggedness, distance to main and secondary roads, and distance to buildings^11,26,37,40^ (Table 1). We standardized all variables. We computed the proportion of forest and the terrain ruggedness index using a moving window of 5 × 5 pixels, with a cell resolution of 25 m, i.e., 125 × 125 m around each location.

**Table 1:** Covariates used to model habitat selection of wolf territories in Scandinavia.

Name	Description	Source
Forest	Proportion of forest	Swedish Corine land cover map Lantmäteriet, Sweden, merged with Northern Research Institute’s vegetation map, Norway (25 × 25 m) resolution)
Elevation	Elevation (m)	DEM 25 × 25 m; Geographical Data Sweden, Lantmäteriet; Norge digital, Statens kartverk, Norway
TRI	Terrain ruggedness index	DEM 25 × 25 m; Geographical Data Sweden, Lantmäteriet; Norge digital, Statens kartverk, Norway^81^
Distance Main road	Distance from nearest main road (m)	(1:100 000, Lantmäteriet,Sweden; N50 kartdata, Statens kartverk, Norway)
Distance Secondary road	Distance from nearest secondary road (m)	(1:100 000, Lantmäteriet,Sweden; N50 kartdata, Statens kartverk, Norway)
Distance Building	Distance from nearest building (m)	(1:100 000, Lantmäteriet,Sweden; N50 kartdata, Statens kartverk, Norway)

As we aimed to capture individual variation in habitat selection patterns, we attempted to compute one single resource selection function (RSF) using generalized linear mixed models^49^ with all individual wolves, including ID of the wolf territory as random intercept, but also random slopes. We followed a five-stages protocol adapted from Bolker ^50^ to avoid the lack of model convergence. Therefore, we: (1) centered and scaled predictors; (2) checked for singularities; (3) recomputed the Hessian calculation with the Richardson extrapolation method; (4) restarted the fit from the original value; and, (5) attempted all available optimizers. Nevertheless, we did not avoid lack of convergence and, as an alternative, we quantified individual variation in habitat selection by conducting independent RSFs for each wolf territory, using the same set of predictor variables for each territory-season^51^. We used an exponential function for each territory with the response variable being the used locations (1: GPS locations) and available locations (0: random locations within the home range):

$${\rm{R}}{\rm{S}}{\rm{F}}={\rm{y}}(0,1)=\exp ({\beta }_{1}{X}_{1}+{\beta }_{2}{X}_{2}+\ldots +{\beta }_{{\rm{n}}}{{\rm{X}}}_{{\rm{n}}})$$

where Xi denotes the collection of covariates i. Coefficients, βi, were estimated using logistic regression^52^, and exp(β_i_) can be interpreted as the odds ratio.

Habitat selection can vary based on behavior, i.e., when wolves travel, rest, or handle prey. For example, wolves can use secondary roads for traveling, but not for resting^40^. Wolves spend about 20% of their time traveling^53^, and distance between consecutive GPS locations allows distinguishing traveling from other behaviors. Thus, we repeated the same RSFs based on the exponential function described above, but only using the GPS locations when individuals were traveling, to analyze if the potential effect of sympatry/allopatry with bears reflected on a particular wolf behavior. We defined traveling GPS locations as when the speed from the previous location was >200 m/hr. This distance is equivalent to the predation study protocol designed by the Scandinavian Wolf Research Project, which defined clusters of GPS locations as the overlap of at least two buffers with radius of 100 m around each location^53^.

To further account for a potential effect of habitat availability in wolf habitat selection, we analyzed if there were functional responses on habitat selection, i.e., if wolves selected differently depending on what was available within their respective home ranges. Using the beta coefficients obtained for each individual and each covariate, we linked the habitat selection pattern of each individual for each covariate (i.e., the selection coefficients βi) with the availability of that covariate. Availability was defined by the characteristics of the random locations within the home range of each individual wolf. The assumption behind this analysis was that, without clear functional responses, i.e., with habitat selection not changing with availability, we could compare the coefficients of selection in relation with a particular factor (i.e., brown bear presence/absence in sympatric/allopatric areas, respectively) even if habitat availability regarding other variables could differ among individuals. This analysis revealed a lack of functional responses in wolf habitat selection (Supplementary file).

As a next step, we performed a Principal Component Analysis (PCA) on the matrix containing the resource selection coefficients of each wolf territory, to visualize the effect of sympatry or allopatry with bears and a potential seasonal effect (winter vs. spring-summer) in wolf habitat selection.

### Relationship between wolf habitat selection and sympatry or allopatry with bears

We used generalized linear mixed models^49^ to investigate the relationship between the habitat selection patterns determined by the RSFs (the scores of the PCA) and whether wolves were sympatric or allopatric with bears. The scores of the main PCA axis were the response variables of our models and the category of wolf territories (sympatric or allopatric) and season (winter or spring-summer) as interacting predictors. If sympatric and allopatric wolf territories showed different habitat selection patterns, we would find a different sign in their estimates for some habitat variables. We conducted this analysis for all GPS locations and then for a subset of the GPS locations of wolves, while traveling. We used the ID of the wolf territory as random intercept with the lmer function and a Gaussian distribution, because some territories were monitored over several seasons/years (Fig. 1). We also included moose density, the wolves’ staple prey, as a covariate in the models. Moose density was extracted from harvest statistics collected at the moose management unit level in Sweden and at the municipality level in Norway, as the average number of moose harvested per km^2^ inside the wolf territory with a time lag (year t + 1)^26,54^. We used Akaike’s Information Criterion (AIC)^55,56^ for model selection, considering models within two AIC units as equally supported^55^. We conducted all analyses with R 3.3.3^57^.

## Results

### Wolf habitat selection

**Figure 2:**
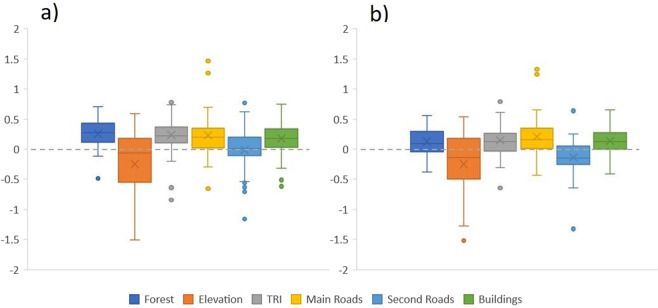
Box plot of the coefficients of selection determined with resource selection functions (RSFs) performed at the wolf territory level for the environmental variables: forest, elevation, rugged terrain (TRI), distance to main and secondary roads, and distance to buildings, using (**a**) all GPS-locations and (**b**) only travelling locations. For each variable, the box shows the 1st and 3rd quartile, the horizontal line is the median and the cross is the mean.

Wolves generally selected for forested and rugged terrain and avoided human-related features of the landscape, i.e., wolves selected for areas further away from main roads and buildings, as illustrated by the estimated coefficients of selection for different habitat variables. This pattern was similar whether using all GPS locations or only the GPS locations of wolves when traveling (Fig. 2). Likewise, the pattern was consistently similar defining habitat availability with MCP (Fig. 2) and kernel methods (Fig. 1a, Supplementary file).

### Relationship between habitat selection and sympatry or allopatry with bears

**Figure 3:**
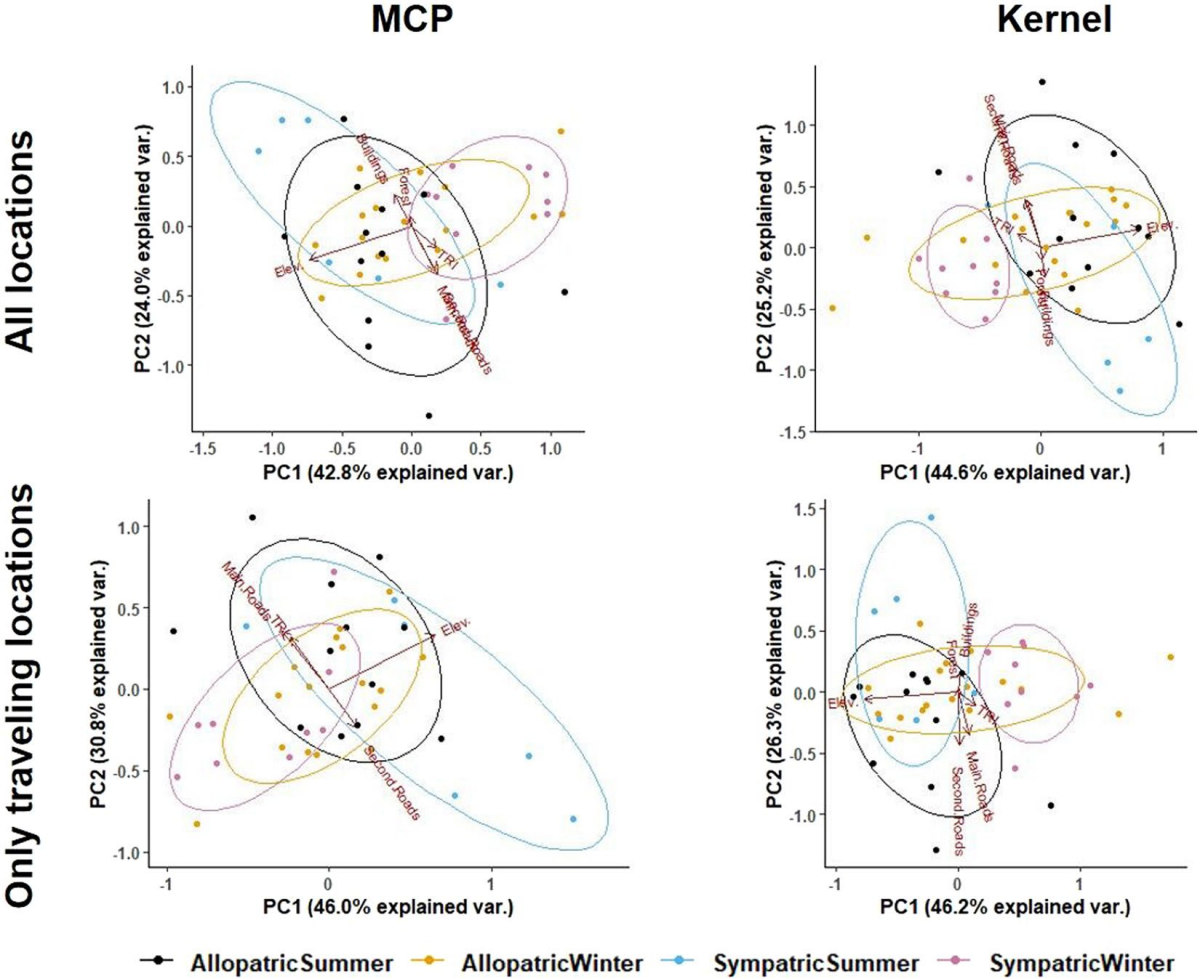
Principal component analysis illustrating wolf habitat selection in Scandinavia in areas sympatric or allopatric with brown bears, using all GPS locations recorded (top panels) and those when wolves were traveling (bottom panels). We used a 100% minimum convex polygon (MCP, left panels) or a 99% kernel home range (right panels) for each wolf territory, to quantify habitat availability. “Elev” is elevation, and all variables are described in Table 1.

The first and second axis of the PCA explained up to 46% and 30%, respectively, of the variation in wolf habitat selection (i.e., ~75% in total, Fig. 3) and were used for further analyses. The PCA showed that wolves with territories in areas sympatric with bears selected for lower elevations in winter. This pattern was similar when using all GPS locations and only those when wolves were traveling, regardless of the alternative definition of habitat availability (MCP or kernel; Fig. 3).

As described above (Methods), the scores of the main PCA axis (PC1 and PC2) were the response variables in the regression models. Elevation was the main variable explaining the variation in PC1 (Fig. 3), and the best models confirmed the negative relationship between elevation and wolf habitat selection in winter (Table 2). Avoidance of higher elevation in winter was more consistent for wolf territories sympatric with bears (i.e., the 95% confidence interval around the estimate never included 0) than for wolf territories allopatric with bears (Table 2), thus reaffirming the pattern of avoidance of higher elevation illustrated by the PCA (Fig. 3). The results were consistent and not dependent on alternative definitions of habitat availability (MCP or kernel) or on the type of GPS-locations used (Table 2). Regarding PC2, whose variation was mostly driven by a combination of natural (forest and rugged terrain) and human-related features of the landscape, the null model was the most supported in all cases (Table A2 in Supplementary file). For both PC1 and PC2, the second-best model in each case (with MCP or kernel; with all GPS or only traveling locations) retained the effect of moose density. However, the 95% confidence interval around the estimate of the moose density effect included 0 in all cases, i.e., the direction of the effect was not clear (Table 2 and Table A2), suggesting that moose density *per se* had relatively little effect on wolf habitat selection, regardless of sympatry or allopatry with bears.

**Table 2:** Output of the generalized linear mixed models to analyze wolf habitat selection in Scandinavia, using the scores of the PC1 as response variable (variation in PC1 was mostly explained by Elevation).

Type of location	Type of Home Range	Model rank	Model Coefficients					df	logLik	AICc	Delta AICc	Model weight
All locations	KERN	1	**Seasons**					6	−36,7	87,6	0	0,60
				**Estimate**	**Lower 95% CI**	**Upper 95% CI**	**t value**					
			(Intercept)	0,36291	0,04687	0,67895	2,297					
			Allopatric Winter	−0,38762	−0,7811	0,00586	−1,97					
			Sympatric Summer	0,05266	−0,50846	0,61378	0,188					
			Sympatric Winter	−0,9522	−1,4583	−0,4461	−3,763					
		2	**Seasons + Moose**					7	−35,8	88,8	1,15	0,34
				**Estimate**	**Lower 95% CI**	**Upper 95% CI**	**t value**					
			(Intercept)	0,43673	−0,15909	1,03255	1,466					
			Allopatric Winter	−0,38642	−0,78402	0,01118	−1,944					
			Sympatric Summer	0,02202	−0,58134	0,62538	0,073					
			Sympatric Winter	−0,98664	−1,54896	−0,42432	−3,509					
			Moose	−0,25475	−2,05037	1,54087	−0,284					
		3	Null Model					3	−43,4	93,3	5,68	0,04
		4	Moose					4	−42,5	93,9	6,33	0,03
	MCP	1	**Seasons**					6	−34,3	82,8	0	0,53
				**Estimate**	**Lower 95% CI**	**Upper 95% CI**	**t value**					
			(Intercept)	−0,2327	−0,5319	0,0665	−1,556					
			Allopatric Winter	0,226	−0,141	0,593	1,232					
			Sympatric Summer	−0,2629	−0,7983	0,2725	−0,982					
			Sympatric Winter	0,7697	0,2837	1,2557	3,168					
		2	**Seasons + Moose**					7	−33,1	83,4	0,57	0,4
				**Estimate**	**Lower 95% CI**	**Upper 95% CI**	**t value**					
			(Intercept)	−0,4383	−0,9983	0,1217	−1,565					
			Allopatric Winter	0,2164	−0,1532	0,586	1,171					
			Sympatric Summer	−0,1832	−0,7498	0,3834	−0,647					
			Sympatric Winter	0,8615	0,3321	1,3909	3,255					
			Moose	0,7463	−0,9443	2,4369	0,883					
		3	Null Model					3	−40,5	87,5	4,71	0,05
		4	Moose					4	−39,8	88,6	13,38	0,001
Only moving locations	KERN	1	**Seasons**					6	−31,5	77,2	0	0,64
				**Estimate**	**Lower 95% CI**	**Upper 95% CI**	**t value**					
			(Intercept)	0,387227	0,102661	0,671793	−2,722					
			Allopatric Winter	0,357956	0,027826	0,688086	2,169					
			Sympatric Summer	0,001058	−0,523912	0,526028	0,004					
			Sympatric Winter	0,954532	0,46789	1,441174	3,923					
		2	**Seasons + Moose**					7	−30,7	78,5	1,24	0,35
				**Estimate**	**Lower 95% CI**	**Upper 95% CI**	**t value**					
			(Intercept)	−0,4504	−1,01538	0,11458	−1,594					
			Allopatric Winter	0,35728	0,02378	0,69078	2,143					
			Sympatric Summer	0,02766	−0,54028	0,5956	0,097					
			Sympatric Winter	0,98447	0,44175	1,52719	3,628					
			Moose	0,21745	−1,51675	1,95165	0,251					
		3	Null Model					3	−39,8	86,2	8,98	0,007
		4	Moose					4	−38,9	86,8	9,58	0,005
	MCP	1	**Seasons**					6	−30,1	74,5	0	0,52
				**Estimate**	**Lower 95% CI**	**Upper 95% CI**	**t value**					
			(Intercept)	0,05595	−0,21069	0,32259	0,42					
			Allopatric Winter	−0,09552	−0,4332	0,24216	−0,566					
			SympatricSummer	0,5935	0,12438	1,06262	2,53					
			Sympatric Winter	−0,44264	−0,86248	−0,0228	−2,109					
		2	**Seasons + Moose**					7	−29	75,1	0,61	0,38
				**Estimate**	**Lower limit**	**Upper limit**	**t value**					
			(Intercept)	0,25888	−0,22414	0,7419	1,072					
			Allopatric Winter	−0,08504	−0,42502	0,25494	−0,5					
			Sympatric Summer	0,51485	0,02347	1,00623	2,096					
			Sympatric Winter	−0,53346	−0,98548	−0,08144	−2,36					
			Moose	−0,7433	−2,18704	0,70044	−1,03					
		3	Null Model					3	−36,01	78,8	4,31	0,06
			Moose					4	−35,22	79,5	5	0,04

Habitat selection was analyzed for wolf territories sympatric or allopatric with brown bears, taking into account seasonality (winter vs spring-summer seasons), moose density, and wolf territory id (random factor). We tested models with two types of wolf GPS locations (using only moving locations in one set of models, and all locations in another set), and two proxies of habitat availability, i.e., building models with MCP and kernel methods (see Methods for further details).

**Figure 4:**
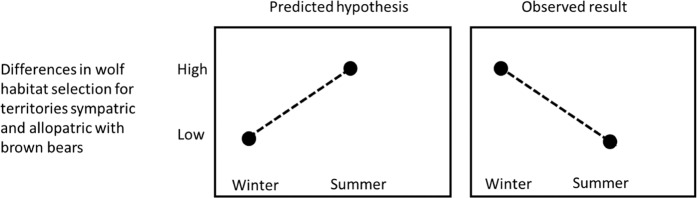
We expected wolf habitat selection to be different when sympatric and allopatric with brown bears in Scandinavia in summer, when bears are active, but not in winter, during bear hibernation, but we found the opposite. Differences in wolf habitat selection when sympatric or allopatric with bears were clearer in winter.

We did not find any other support for different wolf habitat selection within home ranges in areas where wolves were sympatric or allopatric with bears. Our prediction that wolf habitat selection in summer would be different in areas sympatric compared to allopatric with bears was not supported. In contrast, we found that wolf habitat selection differed more clearly in winter between areas sympatric and allopatric with bears (Fig. 4 and Table 2).

## Discussion

We have previously found several indications of an effect of the presence of bears on wolf habitat selection in Scandinavia^26,27^, and that bears and wolves segregate more than expected by chance in their habitat selection^37^. The present study showed that wolves avoided higher altitudes during winter, particularly in the area where wolf territories were sympatric with bears, i.e., in the northern part of the wolf breeding range in Scandinavia. It is important to note, however, that this area differed from the southern part of the wolf breeding range, where wolves and bears were allopatric, in that the northern part is typically characterized by higher elevation. Because all bears hibernate for 5–7 months in winter in Scandinavia^39^, interspecific competition with bears may not be the actual driver of wolf avoidance of higher altitudes in winter, even though it has been documented that male bears prefer higher elevation within the forest range for denning^36^.

In northern Europe, an altitudinal migration of cervids in general, and moose in particular, occurs in winter, with animals moving to lower altitudes to ease movement and increase energy intake^58–60^. Larger altitudinal gradient and moose migration become more common with increasing northern latitude, whereas short altitudinal gradients correspond to shorter horizontal displacement^61^. Altitudinal moose migration has also been documented where bears and wolves are sympatric in Scandinavia^62^. Thus, the general occurrence of moose at lower elevations in winter is likely the mechanism explaining our result of wolf avoidance of high elevations in winter^63^. In our study area, altitude reaches ~1000 m.a.s.l. in the northern part, where wolves and bears are sympatric, whereas most of the wolf territories allopatric with bears are typically found in areas at lower elevations (see Fig. 1 and Methods), with some exceptions on the Norwegian side of the study area.

Wolf kill rates increase with increasing snow depth in some ecosystems, e.g., in Bialowieza (Poland)^64^ and Michigan (USA)^65^. However, snow depth was not a significant predictor of wolf kill rates in Yukon (Canada)^66^ and did not explain variation in hunting success among wolf territories in Scandinavia^67^. Nevertheless, wolves change movement patterns, sociality, and feeding behavior in response to snow-related changes in prey distribution and mobility^68^. Thus, it seems reasonable that wolves’ selection of lower altitudes during winter is a result of moose redistribution^69^, because moose is the main prey in the northern part of the Scandinavian wolf range^70^.

We have previously found that wolves and bears segregate more than expected by chance within home ranges where both species are sympatric in Scandinavia, particularly in late spring and early summer, when both predators rely on neonate moose calves^37^. Nevertheless, in that study we found both similarities and differences in wolf and bear habitat selection. In particular, wolves, an obligate carnivore, selected for habitats with higher moose densities than bears did^37^. The observed wolf avoidance of higher altitudes in winter in this present study seems to reinforce the tight relation between wolf habitat selection and the habitat selection of their staple prey, moose, which in Scandinavia occur in high densities compared to other areas^67^.

In Scandinavia, wolves and bears compete seasonally for the same resource, i.e., neonate moose calves, which are born and heavily preyed upon by both predators in spring and early summer. This competition could affect habitat selection^37^ and wolf kill rates, which are lower in areas sympatric with bears than in allopatric areas^38^. In the present study, and against our prediction (Fig. 4), we found no differences in wolf habitat selection in spring-summer for wolf territories sympatric or allopatric with bears as an apparent effect of interspecific interaction between these apex predators. This indicates that a given driver of habitat selection can have varying influence at different spatial scales^30^, and highlights the importance of seasonality to understand interspecific interactions^31,37^.

Regardless of sympatry or allopatry with bears, wolves generally selected for forested and rugged terrain and avoided human-related features of the landscape. This general pattern was very similar when using all GPS locations or only those recorded while traveling, and with different definitions of habitat availability (MCP/kernel; Fig. 2 and Fig. 1a, respectively). This is in line with previous findings for this and other large carnivore populations, e.g., in Scandinavia^26,27^, and suggests that our variables were adequate to find a pattern of wolf habitat selection in agreement with previously published literature. However, this does not necessarily mean that our variables represented all factors that can influence wolf habitat selection. The very large spatial and temporal scale of our study, performed across ~100,000 km^2^ and a 15–year time span, may imply that some unmeasured factors masked or overrode the potential effect of interspecific competition with bears at this large scale, for instance. It is noteworthy, however, that forest cover and road and building densities did not change over the study period and, most remarkably given the goal of the study, bear density has been stable since the 1990’s in the study area^26^, i.e., the variables we included were robust. Intraspecific factors, such as wolf density, also drive wolf habitat selection^26^; e.g., the effect of natal habitat imprinting on wolf habitat selection during the dispersal phase and later in life has recently been documented^27,71^.

Human avoidance is indeed an overwhelming factor explaining large carnivore habitat selection in human-dominated landscapes, and wolves are no exception^26,72,73^. Although bear density has a negative effect on the probability of wolf pair establishment in Scandinavia, avoidance of main roads is even more important^26^. Likewise, both bears and wolves avoid human-related habitats within their home ranges during daytime^37^.

In this study, wolves generally selected against human-related features of the landscape throughout the study area and against higher elevations in winter, particularly in the northern area, where bears and wolves overlap. The latter result may be explained by moose seasonal distribution and the typically higher elevation in the northern part of our study area, which results in a deeper snow cover in winter than in the southern part of the area^74^. Nevertheless, we suggest that it is worth highlighting the value of the apparent lack of an effect of the presence of bears on habitat selection by territorial, adult wolves in our present study. Communication or publication of scientific results is easier when results are positive and novel than when they are negative, confirmatory, or merely supportive of null hypotheses^75^. However, negative results can lead researchers to critically evaluate and validate their way of thinking^76^. In this context, the lack of an effect of bears in our study, in contrast to previous results, illustrates empirically that a given driver of habitat selection can play a different role at different spatial and seasonal scales contexts, as suggested earlier^30^. Because of the scarcity of studies on interspecific competition between large carnivores at the population level, finding a varying effect of interspecific competition, i.e., a varying effect of bear presence on wolf habitat selection at different spatial and temporal context, is relevant to understanding the complex structure of ecological communities and mechanisms underlying interactions.

Accounting for availability is crucial in habitat selection studies^77^. In this study, we compared the habitat selection pattern of individuals whose available resources varied depending on where they had established their territories. As shown in the Supplementary file, we found no evidence for the presence of functional responses in the wolves’ habitat selection in relation to habitat availability^78^. This means that individuals did not seem to change their behavior according to resource availability. Without functional responses, i.e., with habitat selection not changing with availability, we suggest we can compare the coefficients of selection in relation with a given factor (namely, brown bear presence/absence in sympatric/allopatric areas, respectively), even if habitat availability regarding other variables differed among individuals.

Finally, most of the individual wolf-bear interactions described in the literature have been observed at carcasses^79^, and fine-scale movements around kill sites may be another mechanism used to reduce the risk of encounters and interactions between sympatric predators^37^. Thus, to complement studies at second and third order of habitat selection, including previous studies^26,27,37^ and this one, we emphasize that future studies should explore the potential effect of interspecific interactions at the fourth order of habitat selection and in relation with other behaviors. For instance, analyzing the potential effect of interspecific interactions at resting and kill sites in areas sympatric or allopatric with brown bears in general, and with different sex and age classes of bears in particular. Although rare, remains of puppies and cubs of wolves and bears, respectively, have been found in scats of the other predator^79,80^, thus analyzing potential interactions at breeding dens of both species and at rendezvous sites of wolves could also be interesting. Such studies would provide further knowledge of the potential effect of interactions at finer spatial scales and in relation with specific behaviors, such as resting, foraging, and breeding.

## Data availability

Data would be made available on Dryad Digital Repository.

## Supplementary information

Supplementary file.
