# Supplementary material for: Wolf habitat selection when sympatric or allopatric with brown bears in Scandinavia
Source: Sci Rep. 2020 Jun 18;10:9941. doi: 10.1038/s41598-020-66626-1 (PMC7303184; doi:10.1038/s41598-020-66626-1)
Supplement: Supplementary file 1 — Supplementary file. [file 41598_2020_66626_MOESM1_ESM.pdf]

Wolf habitat selection when sympatric or allopatric with brown bears in Scandinavia

Andrés Ordiz[1,2,3*a], Antonio Uzal[3,a], Cyril Milleret[2], Ana Sanz-Pérez[4], Barbara Zimmermann[1], Camilla Wikenros[5], Petter Wabakken[1], Jonas Kindberg[6,7], Jon E Swenson[2], and Håkan Sand[5]

[1]Faculty of Applied Ecology, Agricultural Sciences and Biotechnology, Inland Norway University of Applied Sciences, Evenstad, NO-2480 Koppang, Norway

[2]Faculty of Environmental Sciences and Natural Resource Management, Norwegian University of Life Sciences, Postbox 5003, NO–1432 Ås, Norway

[3]School of Animal, Rural and Environmental Sciences, Nottingham Trent University, Brackenhurst, Southwell, Nottinghamshire NG25 0FQ, UK

[4]Biodiversity and Animal Conservation Lab, Forest Science and Technology Centre of Catalonia (CTFC), 25280 Solsona, Spain

[5]Grimsö Wildlife Research Station, Department of Ecology, Swedish University of Agricultural Sciences, SE-730 91 Riddarhyttan, Sweden

[6]Norwegian Institute for Nature Research, NO–7485 Trondheim, Norway

[7]Department of Wildlife, Fish, and Environmental Studies, Swedish University of Agricultural Sciences, SE–901 83 Umea, Sweden

[a] Both authors contributed equally to this manuscript.

[*] **Corresponding author:**

Andrés Ordiz

andres.ordiz@gmail.com; Phone: +34689987062

Fig. 1A. Box plot of the coefficients of selection determined with resource selection functions (RSFs) performed at the wolf territory level for the environmental variables: forest, elevation, rugged terrain (TRI), distance to main and secondary roads, and distance to buildings, using a) all GPS-locations and b) only traveling locations. For each variable, the box shows the 1st and 3rd quartile, the horizontal line is the median and the cross is the mean. Habitat availability was defined using minimum convex polygons for each wolf territory (Fig. 2 in the main text) and kernel techniques (this figure). Both approaches yielded the same result.

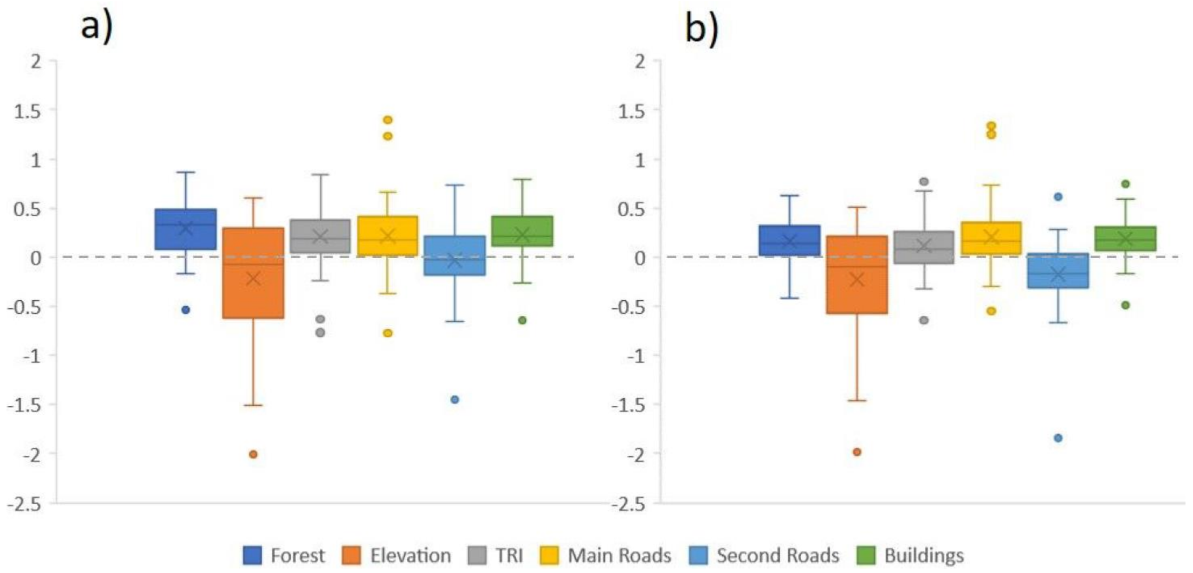

Functional responses

We checked if there were functional responses on habitat selection, i.e., if wolves selected differently depending on what was available within their respective home ranges. Using the beta coefficients obtained for each individual and each covariate, we linked the habitat selection pattern of each individual for each covariate (i.e., the selection coefficients βi) with the availability of that covariate. Availability was defined by the characteristics of the random locations within the home range of each individual wolf. This analysis revealed a lack of functional responses in wolf habitat selection, regardless of defining home ranges with kernel or 100% minimum convex polygon (MCP) techniques and using all wolf locations or only traveling locations (see Methods for a full description). In all panels below, blue lines are the linear regression and smooth local regression lines. The gray shade indicates the 95% confidence interval for each of these methods of fitting a line, and asterisks represent the selection coefficients βi for each covariate and individual wolf.

With Kernel, all GPS locations:

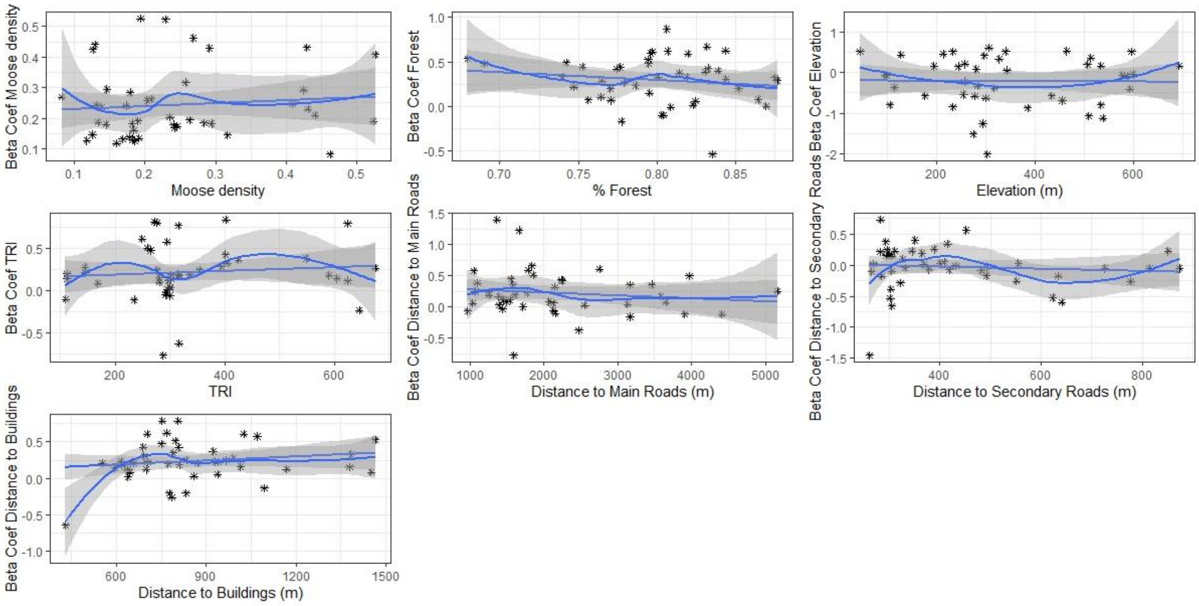

With MCP, all GPS locations:

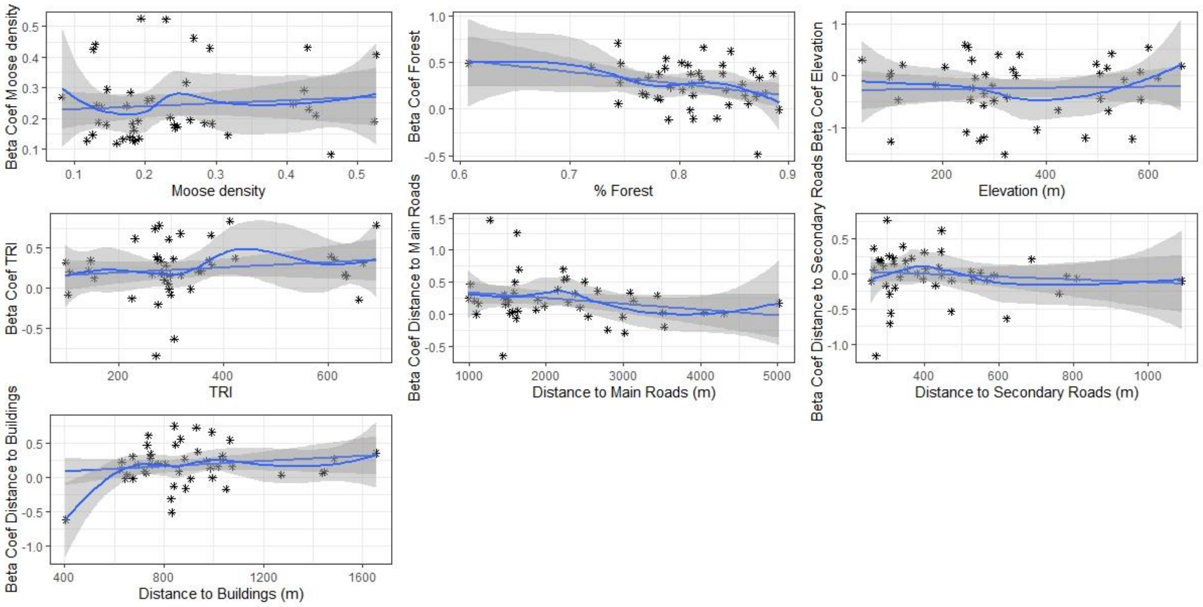

With Kernel, only moving GPS locations:

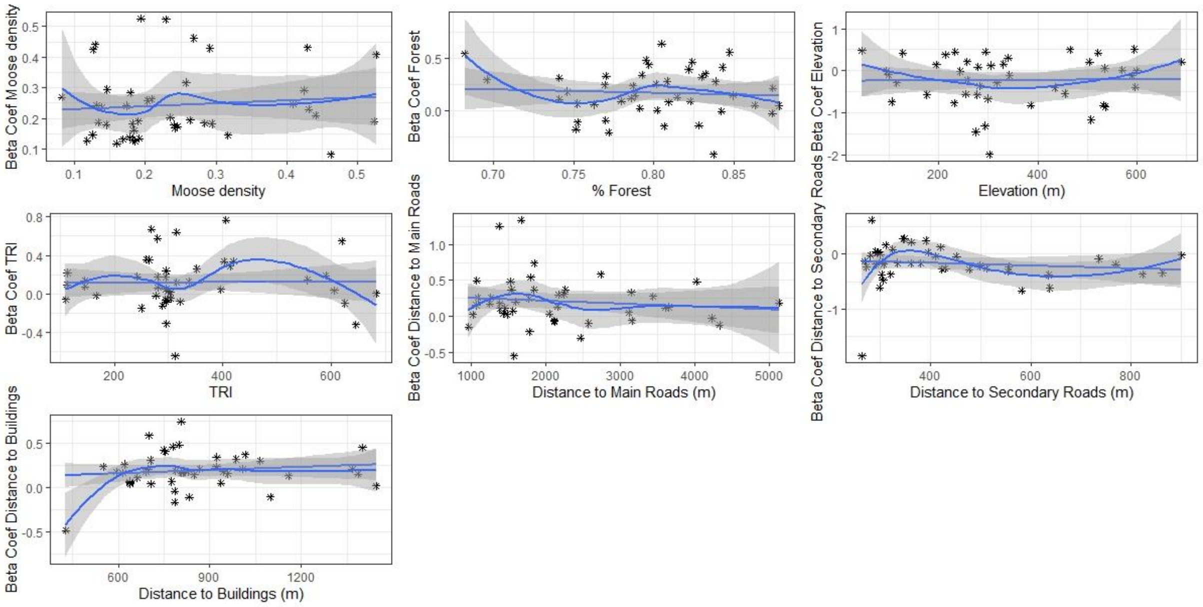

With MCP, only moving GPS locations:

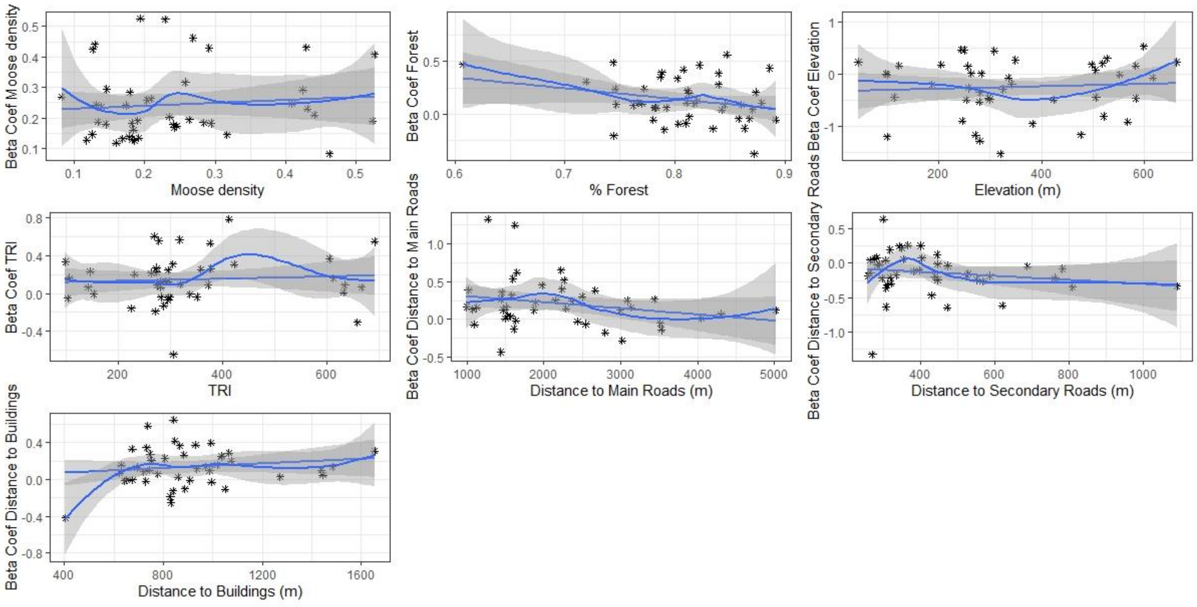

Table A1. Summary information on the resulting environmental data at the locations used by wolves and at available locations, for each of the methods we used to define habitat availability (MCP and Kernel methods, see main text).

Variable	MCP all locations				MCP only moving locations			
	Used		Available		Available		Available	
	Mean	SD	Mean	SD	Mean	SD	Mean	SD
Moose	0.245	0.113	0.245	0.113	0.233	0.102	0.233	0.102
Human	<0.001	0.014	0.003	0.042	0.001	0.019	0.002	0.041
Agri	0.006	0.062	0.019	0.116	0.009	0.078	0.017	0.109
Forest	0.873	0.272	0.807	0.335	0.851	0.292	0.808	0.335
Mires	0.077	0.207	0.092	0.229	0.085	0.217	0.092	0.229
Water	0.036	0.162	0.068	0.233	0.045	0.183	0.067	0.230
Mountains	0.007	0.079	0.011	0.095	0.009	0.085	0.013	0.106
TRI	414.596	392.965	344.952	315.523	398.400	386.463	353.696	328.460
Altitude	327.247	175.834	332.988	186.833	343.181	182.678	348.796	190.145
Main road	2277.619	1835.489	2113.307	1932.276	2396.272	1968.857	2229.659	2031.414
Secondary road	414.531	405.949	420.873	464.567	405.148	433.956	433.886	496.645
Building	956.370	585.432	850.694	597.802	941.855	590.091	861.987	603.741

Variable	Kernel all locations				Kernel only moving locations			
	Used		Available		Available		Available	
	Mean	SD	Mean	SD	Mean	SD	Mean	SD
Moose	0.245	0.113	0.245	0.113	0.233	0.102	0.233	0.102
Human	<0.000	0.014	0.004	0.053	0.001	0.019	0.004	0.050
Agri	0.006	0.062	0.025	0.133	0.009	0.078	0.022	0.127
Forest	0.873	0.272	0.802	0.339	0.851	0.292	0.801	0.340
Mires	0.077	0.207	0.089	0.225	0.085	0.217	0.091	0.227
Water	0.036	0.162	0.069	0.233	0.045	0.183	0.068	0.232
Mountains	0.007	0.079	0.012	0.099	0.009	0.085	0.014	0.109
TRI	414.596	392.965	343.214	302.767	398.400	386.463	348.926	311.553
Altitude	327.247	175.834	333.695	189.967	343.181	182.678	350.460	193.572
Main road	2277.619	1835.489	2119.942	2018.913	2396.272	1968.857	2243.488	2114.287
Secondary road	414.531	405.949	416.891	462.496	405.148	433.956	431.199	489.584
Building	956.370	585.432	813.422	582.822	941.855	590.091	828.460	590.615

Table A2. Output of the generalized linear mixed models to analyze wolf habitat selection in Scandinavia, using the scores of the PC2 as response variable (variation in PC1 was mostly explained by natural (forest and rugged terrain) and human-related features of the landscape). Habitat selection was analyzed for wolf territories sympatric or allopatric with brown bears, taking into account seasonality (winter vs spring-summer seasons), moose density, and wolf territory id (random factor). We tested models with two types of wolf GPS locations (using only moving locations in one set of models, and all locations in another set), and two proxies of habitat availability, i.e., building models with MCP and kernel methods (see Methods for further details).

Type of location	Type of Home Range	Model rank	Model Coefficients	Estimate	Lower 95% CI	Upper 95% CI	t value	df	logLik	AICc	Delta AICc	Model weight
All locations	KERN	1	Null Model					3	-28.69	64.0	0	0.61
		2	Moose					4	-27.98	65.0	1.02	0.37
			(Intercept)	-0.0157	-0.4283	0.3968	-0.0750					
			Moose	0.2950	-1.1722	1.7621	0.3940					
		3	Seasons					6	-28.45	71.2	7.19	0.02
		4	Seasons+Moose					7	-27.73	72.6	8.59	<0.01
	MCP	1	Null Model					3	-26.78	60.2	0	0.65
		2	Moose					4	-26.23	61.5	1.32	0.34
			(Intercept)	-0.0083	-0.3816	0.3650	-0.0430					
			Moose	-0.1461	-1.4821	1.1899	-0.2140					
			Seasons					6	-27.26	68.8	8.63	0.01
			Seasons+Moose					7	-26.57	70.2	10.08	<0.01
Only Moving Locations	KERN	1	Null Model					3	-25.68	58.0	0	0.58
		2	Moose					4	-25.11	59.3	1.30	0.30
			(Intercept)	-0.0624	-0.4527	0.3279	-0.3140					
			Moose	0.0832	-1.3031	1.4696	0.1180					
			Seasons					6	-23.95	62.2	4.21	0.07
			Seasons+Moose					7	-23.11	63.3	5.38	0.04
	MCP	1	Null Model					3	-25.54	57.7	0.00	0.64
		2	Moose					4	-25.00	59.0	1.36	0.32
			(Intercept)	0.0338	-0.3411	0.4086	0.1770					
			Moose	0.0671	-1.2695	1.4037	0.0980					
			Seasons					6	-25.13	64.5	6.86	0.02
			Seasons+Moose					7	-24.36	65.8	8.17	0.01
